# miR-6126 modulates GRP78 to suppress the Warburg effect and mitochondrial dynamics in triple-negative breast cancer

**DOI:** 10.7150/ijms.107240

**Published:** 2025-07-28

**Authors:** Wei-Jan Wang, Jian-Sheng Liu, Nguyen Hoang Anh Kha, Wan-Jou Shen, Chih-Jung Chen, Shuan-Chu Chen, Pin-Chen Liao, Chih-Yang Huang, Wei-Wen Kuo

**Affiliations:** 1 Department of Biological Science and Technology, College of Life Sciences, China Medical University, Taichung, Taiwan ROC.; 2 Cancer Biology and Precision Therapeutics Center and Research Center for Cancer Biology, China Medical University, Taichung, Taiwan ROC.; 3 Thoracic Department, China Medical University Beigang Hospital, Yunlin, Taiwan ROC.; 4 Graduate Institute of Biomedical Sciences, China Medical University, Taichung, Taiwan ROC.; 5 Surgical Department, China Medical University Hospital, Taichung, Taiwan ROC.; 6 Cardiovascular and Mitochondrial Related Disease Research Center, Hualien Tzu Chi Hospital, Buddhist Tzu Chi Medical Foundation, Hualien, Taiwan ROC.; 7 Center of General Education, Buddhist Tzu Chi Medical Foundation, Tzu Chi University of Science and Technology, Hualien, Taiwan ROC.; 8 Department of Medical Research, China Medical University Hospital, China Medical University, Taichung, Taiwan ROC.; 9 Department of Medical Laboratory Science and Biotechnology, Asia University, Taichung, Taiwan ROC.; 10 Program for Biotechnology Industry, China Medical University, Taichung, Taiwan ROC.

**Keywords:** Warburg effect, miR-6126, GRP78, triple-negative breast cancer, mitochondria dynamics

## Abstract

Cancer cells often exhibit a metabolic shift towards aerobic glycolysis, known as the Warburg effect, leading to excessive energy production that facilitates tumorigenesis, including in breast cancer. Recently, non-coding RNAs, including microRNAs (miRNAs), have been identified as playing crucial roles in various human cancers. However, their roles in regulating metabolic reprogramming in breast cancer remain largely unexplored. Here, we identified the novel miRNA miR-6126, which is highly expressed in TNBC cells by using a miRNA microarray analysis. Overexpression of miR-6126 reduced the growth of TNBC cells and induced apoptosis by targeting GRP78 *in vitro* and* in vivo*. In addition, a luciferase reporter assay confirmed that GRP78 is a direct target of miR-6126. Elevated glucose metabolism, indicated by increased levels of LDHA and glucose transporter-1, was observed in TNBC following GRP78 overexpression. Treatment with miR-6126 mimics or GRP78 siRNA not only reduced LDHA and GLUT1 expression but also decreased glucose uptake and lactate release in TNBC cells. Moreover, miR-6126 impaired mitochondrial function by inducing mitochondrial fission through the downregulation of phospho-Drp1 (Ser616) and FIS1. Furthermore, we demonstrated that the expression of miR-6126 is negatively correlated with GRP78 in human tumor tissues. Our findings revealed that miR-6126 is implicated in tumorigenesis via the Warburg effect by targeting GRP78 and restoring mitochondrial function in TNBC.

## Introduction

Breast cancer is the most frequently diagnosed malignancy among women worldwide, and it is the second leading cause of cancer-related death in women 1. Breast cancer is classified into different subtypes, which have varying therapeutic sensitivities and clinical prognoses. Triple-negative breast cancer (TNBC) is characterized by the absence of estrogen receptor (ER-), progesterone receptor (PR-), and human epidermal growth factor receptor 2 (HER2-). TNBC is the most invasive subtype, represents approximately for 15-20% of all breast cancer cases. Less than 30% of metastatic TNBC patients survive for more than 5 years 2. Surgery followed by radiotherapy and chemotherapy is the standard treatment protocol for breast cancer management, with the aim of reducing the likelihood of cancer recurrence 3. However, as TNBC lacks the expression of hormones, endocrine therapy is out of the option, making chemotherapy the only treatment against TNBC 4. Therefore, investigating the molecular processes necessary for TNBC development could be crucial for enhancing the effectiveness of patient treatment.

MicroRNAs (miRNA) increased in cancer cells are termed oncogenes, and conversely, tumor suppressors are those scarce in cancer tissue. Recent publications on the vital role of miRNAs in cancer treatment are growing 5, 6. For instance, a previous study discovered that enhanced miR-6162 expression inhibits multiple oncogenic functions such as proliferation, angiogenesis, invasion, and migration in ovarian cancer 7. Moreover, recent research has highlighted the potential of miRNAs in distinguishing the molecular subtypes of TNBC and developing reliable diagnostic tools. Several studies indicate that miRNAs could serve as effective biomarkers for TNBC and as drivers of tumorigenesis involved in TNBC 8. Furthermore, the expression of different miRNAs has been suggested to differentiate TNBC from other clinical breast cancer subtypes. For example, previous study demonstrated that miR-200c expression levels were consistently lower in TNBC cell lines compared to other breast cancer types, and the results showed the suppression of cell proliferation and invasion by HDAC inhibitors through the regulation of miR-200c expression 9. In addition, another research also revealed that the inhibition of miR-29b-3p could suppress proliferation and induce apoptosis in TNBC 10. Moreover, recent studies have highlighted the involvement of microRNAs (miRNAs) in endocrine resistance. One such example is miR-205, which is significantly downregulated in tamoxifen-resistant breast cancer cells. Functional analyses have demonstrated that miR-205 directly targets Mediator Subunit 1 (MED1), an estrogen receptor-α (ERα) coactivator implicated in tamoxifen resistance 11. In addition, miRNAs have also been shown to play important roles in resistance to anticancer chemotherapy. For instance, recent studies have reported that miR-221 promotes tamoxifen resistance by downregulating cell cycle inhibitors such as p27 in breast cancer cells 12. These advancements hold promise in addressing miRNA associated with TNBC, providing improved treatment strategies by targeting miRNA.

In this study, by using miRNA microarray to compare TNBC and normal breast epithelial cell lines, we identified that miR-6126 shows significantly lower expression in TNBC. Furthermore, inducing the expression of miR-6126 in TNBC inhibits the Warburg effect by altering cancer glucose metabolism and restores mitochondrial function, counteracting tumorigenesis in TNBC. This study elucidates the miRNA-mediated regulatory pathways involved in glucose metabolic reprogramming in TNBC, aiming to inhibit tumor progression.

## Materials and Methods

### Animal investigations

All operations mice followed the protocol established by the National Laboratory Animal Center (NLAC). Subsequently, the rats will be transferred to the China Medical University animal center. Approval for all protocols was obtained from the Institutional Animal Care and Use Committee of China Medical University, and they adhered to the Guide for the Care and Use of Laboratory Animals published by the US National Institutes of Health.

### Cell culture and transient transfection

The normal breast epithelial cell line MCF10A and the triple-negative breast cancer cell line MDA-MB-231 were obtained from the American Type Culture Collection (ATCC, Manassas, VA, USA). MCF10A cells were cultured in DMEM/F12 medium supplemented with EGF (20 ng/mL), hydrocortisone (0.5 mg/mL), cholera toxin (100 ng/mL), insulin (10 μg/mL), 5% horse serum, and 100-1 units/mL penicillin and 100µg/mL streptomycin. MDA-MB-231 cells were cultured in Dulbecco's modified essential medium (DMEM) supplemented with 10% fetal bovine serum, 2 mM glutamine, 100-1 units/mL penicillin, and 100µg/mL streptomycin. All cultures were maintained at 37 °C in humidified air with 5% CO_2_. The cells were grown to 80% confluence on the day of transfection using transfection reagent. Plasmids and siRNAs were transfected following the manufacturer's instructions provided in the jetPRIME® in vitro DNA & siRNA transfection reagent protocol (Polyolus-transfection S.A, New York, NY10020, USA). GRP78 siRNA was purchased from Sigma (St. Louis, MO, USA).

### MicroRNA microarray

The changes in miRNA expression profiles were measured using the Human miRNA OneArray by Phalanx Biotech Group. Hybridization and scanning of the MCF10A and MDA-MB-231 cell lines were conducted following the Phalanx miRNA Hybridization Protocol.

### Transfection with miRNA mimics or mimics negative control

Chemically modified sense RNA (hsa-miR-6126 mimics) or a miRNA mimics negative control (NC) was synthesized by BIOTOOLS Company. Transfection with the miR-6126 mimic was performed using the jetPRIME® in vitro DNA & siRNA transfection reagent protocol according to the manufacturer's instructions (Polyolus-transfection S.A, New York, NY10020, USA). Briefly, 20 nM or 40 nM of miR-6126 mimics or mimics negative control was mixed with 8 μL jetPRIME® in 200 μL jetPRIME® buffer for 1 min at room temperature to form transfection complexes. The cells were incubated with the transfection complexes for 48 hours.

### Cell viability - MTT assay

To assess cell viability, we initially seeded 3x10^4^ cells in 24-well plates. The next day, we transfected the seeded cells with negative controls or miR-6126 mimics for 24, 48, and 72 hours. Subsequently, 500 µl of 3-(4,5-dimethylthiazol-2-yl)-2,5-diphenyl tetrazolium bromide (MTT) solution (5 mg/ml) was added to each well, followed by incubation at 37°C for 2 hours. Afterward, 300 µl of dimethyl sulfoxide (DMSO) was added to replace the supernatant, and the mixture was oscillated for 30 minutes. Absorbance at 540 nm was measured using a microplate reader (Biotech). All results were normalized to the corresponding controls, and the percentage of control was calculated.

### Protein extraction and Western blot analysis

Total protein lysates were prepared using RIPA buffer supplemented with 1% phenylmethylsulfonyl fluoride (PMSF). Protein concentration was determined using the Biorad protein quantification protocol. Subsequently, 30 µg of protein sample was separated by SDS-PAGE (8%, 10%, 12%, 13.5% gel) and transferred onto a PVDF membrane (Millipore, Bedford, MA. 0.45 μm pore size) using a transfer apparatus (Biorad). Nonspecific protein binding was blocked in blocking buffer (5% milk, 20 mM Tris-HCl, pH 7.6, 150 mM NaCl, and 0.1% Tween 20). Primary antibodies, including GRP78, AKT1 (B-1), p-AKT1/2/3 (Ser473), Bad (C-7), p-Bad (Ser136), Bcl-2 (C-2), Bcl-xL (H-5), Bax (P-19), Cytochrome C (7H8), PDH-E1β (17A5), PDK(H-300), Glut4 (N-20), β-actin (C4), and GAPDH (FL-335) (Santa Cruz Biotechnology, Santa Cruz, CA, USA), NFκB p65, Glut1 (Abcam, UK), HIF-1α (Gentex Corporation, USA), PTEN, LDHA (C4B5), HSP70, Caspase 3, cleaved caspase-3 (Asp175) (5A1), p-NF-κB p65 (Ser536) (93H1) (Cell Signaling Technology, USA), were diluted 1:1000 or 1:2000 in antibody binding buffer and incubated overnight at 4°C. The immunoblots were washed three times in TBS buffer for 10 minutes and then incubated in a second solution containing goat anti-mouse IgG-HRP, goat anti-rabbit IgG-HRP, rabbit anti-goat IgG-HRP (Santa Cruz Biotechnology, Santa Cruz, CA, USA), diluted 1:5000 in TBS buffer for 1 hour at room temperature. The immunoblots were then washed in TBS buffer for 10 minutes three times. To quantify protein expression, the protein expression was normalized to GAPDH and β-actin levels.

### RNA extraction and reverse transcription-polymerase chain reaction (Real-time PCR)

Total cellular RNA from cultured cells was isolated using the Direct-zol™ RNA MiniPrep kit (Zymo Research Corporation, USA) following the manufacturer's instructions. RNA samples (2 μg/ml) were then reverse-transcribed into cDNA with a sequence of miR-6162 - 5'-GTGAAGGCCCGGCGGAGA-3', hGRP78-F - 5'-AAGACGGGCAAAGATGTCAG-3' hGRP78-R - 5'-CCGAGTCAGGGTCTCAGAAA-3'. The levels of these miRNA expressions were then normalized to U6 and calculated using the inverse log of the ΔΔCT method. All procedures were conducted following the manufacturer's instructions. The RT-PCR reaction was performed using a standard LightCycler® 480 SYBR Green I Master protocol on a LightCycler® 96 system (Roche). Each 10 μL PCR reaction contained 2 μL of RT product, 2 μL of 5x SyberGreen PCR Mix, 0.3 μL of 10 μM forward primer, 0.3 μL of 10 μM reverse primer, and 5.4 μL of ddH_2_O. Reactions were incubated in a 96-well plate at 95°C for 10 minutes, followed by 40 cycles of 95°C for 10 seconds, 55°C for 15 seconds, and 72°C for 20 seconds. All reactions were performed in triplicate. The cycle number at which the reaction crossed the threshold cycle (Ct) was determined for each gene, and the relative amount of each miRNA to U6 rRNA was calculated using the equation 2ΔCt, where ΔCt = (CtmiRNA - CtU6 rRNA).

### Luciferase assay

The dual luciferase reporter gene assay was conducted to confirm whether HSPA5 was a direct target gene of miR-6126. The full-length sequence of HSPA5 at the 3'-UTR regions. Forward: 5'-CTCGAGATGAAGCTCTCCCTG-3'; Reverse: 5'-AAGCTTCTCATCTTTTTCTGCTGT-3'. The sequence was cloned and inserted into the pmirGLO vector (E1330, Promega Corporation, WI, USA), resulting in the luciferase vector named pHSPA5-WT. Site-directed mutagenesis was performed using bioinformatics software to predict the binding site of miR-6126 and its target gene HSPA5. The pHSPA5-MUT vector was generated using PfuUltra II Fusion HS DNA Polymerase (Agilent Technologies, US). (F: -5'-TGGACCCAATCCCACACACAAGTGTAGATTTTACAAGATAAA-3'; R: -5'-GGGTCAACAATTTATCTTGTAAAATCTACACTTGTGTGTGGGA-3'). Cells were co-transfected with 50 nM miR-6126 mimics and 100 ng luciferase reporter constructs, including the full 3'UTR of GRP78 and the mutated construct, in a 6-well plate using the jetPRIME® in vitro DNA & siRNA transfection reagent protocol according to the manufacturer's instructions (Polyolus-transfection S.A, New York, NY10020, USA). After transfection for 24 hours, cells were lysed, and Firefly luciferase activities were detected using the Dual-Luciferase Reporter Assay System following the manufacturer's instructions (Promega, Madison, Wisconsin, USA). Briefly, 100 µl of luciferase substrate was added to 20 µl of lysate, and luciferase activity was measured using an LB940 Multilabel Reader (Berthold Technologies, Bad Wildbad, Germany). Each luciferase assay was performed in triplicate.

### Confocal microscopy

Cells mounted on glass slides were fixed with 4% paraformaldehyde (PFA) (Sigma) in PBS for 15 minutes at room temperature, then washed three times with PBS. Afterward, the cells were permeabilized with PBS containing 0.1% Triton X-100 and 0.1 mol/L glycine for 15 minutes and blocked with 5% goat serum (GS) in PBS for 30 minutes at room temperature. Subsequently, the cells were incubated with primary antibodies against GRP78, TOM20, COX IV, or Mnf-1 (1:100) at 4°C in 1% GS in PBS overnight. After three washes with PBS, the cells were incubated with Alexa Fluor® 488 and Alexa Fluor® 594 (Invitrogen) at room temperature for 2 hours. Finally, the cells were counterstained with 4',6-diamidino-2-phenylindole (DAPI) (1:10000 dilution) to visualize the nuclei.

### Apoptosis assay

Cells were analyzed for phosphatidylserine exposure using an Annexin-V FITC/propidium iodide double-staining method. The cells were seeded onto a 6 cm dish and, 48 hours later, transfected with miR-6162 mimics or siGRP78. Three different control groups were utilized: unstained cells, cells treated with H_2_O_2_ for 30 seconds and stained with PI (without FITC), and cells treated with H_2_O_2_ for 30 seconds and stained with FITC (without PI). The treatment groups were stained with both FITC and PI. The cells were washed twice with cold PBS and then resuspended in 1x Binding buffer at a concentration of 1x10^6^ cells/ml. Subsequently, 100 μl of the solution was transferred to a 5 ml culture tube, followed by staining with FITC Annexin V and PI, and incubated for 15 minutes at room temperature in the dark. Afterward, 400 μl of 1x Binding Buffer was added to each tube. The samples were then analyzed by flow cytometry.

### Immunohistochemical (IHC) staining and in situ hybridization of tissue microarrays

Breast cancer tissue microarrays, sourced from TissueArray, were analyzed to explore GRP78 and miR-6126 expression using IHC staining and in situ hybridization. The microarrays comprised samples representing 50 TNBC patients. We utilized a GRP78 antibody (Santa Cruz) and custom-designed miRNA-specific probe, acquired from BioTnA Biotech (Taipei, Taiwan), for the IHC staining and hybridization studies. The IHC staining was performed following manufacture protocol 13, 14. The miR-6128 expression level was detected by using the Biospot Kit, strictly following the manufacturer's protocols. Expression levels of the targeted miRNA and GRP78 were quantified with Olympus (OlyVIA V4.1) and the results were normalized to calculate the distribution and intensity of the miR-6126 and GRP78 signals within the tissue sections.

### Lactate production

The lactate concentration of the culture medium was measured using the Lactate Colorimetric/Fluorometric Assay Kit (BioVision, CA, USA) following the manufacturer's instructions. Briefly, MDA-MB-231 cells were transfected with miRNA negative control, miR-6126, or GRP78 siRNA, and the culture medium was collected after 48 hours. The culture medium was mixed with lactate assay buffer at a ratio of 50 μL per well in a 96-well plate. Then, 50 μL of reaction buffer was added to each well and incubated for 30 minutes at room temperature. The lactate production was measured by absorbance (570 nm) using a microplate reader.

### Glucose uptake

Glucose uptake was measured using the 2-NBDG Glucose Uptake Assay Kit (Cell-Based) (BioVision, CA, USA) following the manufacturer's instructions. 2-NBDG (2-deoxy-2-[(7-nitro-2,1,3-benzoxadiazol-4-yl) amino]-D-glucose) is a fluorescent deoxyglucose analog that can be taken up by cells through glucose transporters. After treatment, cells were incubated with 2-NBDG Reagent and Glucose Uptake Enhancer in DMEM medium (0.5% FBS) at 37°C with 5% CO_2_ for 30 minutes. To use Phloretin as a control, cells were treated with 1x Phloretin in DMEM with 0.5% FBS at 37°C with 5% CO_2_ for 1 hour. After incubation, cells were collected from the plate and kept on ice. They were then washed once with 1 ml ice-cold 1x Analysis Buffer and spun down at 400 xg for 5 minutes. The cell pellet was resuspended in 400 μl of 1x Analysis Buffer. The cells were ready to be analyzed using a flow cytometer (488 nm excitation laser) and a fluorescence microscope (excitation range 420 nm-495 nm).

### Seahorse analysis

MB231 cells were transfected with siGRP78 (50 nM) and miR-6126 mimic (40 nM) and the oxygen consumption rate (OCR) was measured using a Seahorse XF96 analyzer (Seahorse Biosciences). Briefly, MDA-MB-231 cells were seeded at a density of 20,000 cells per well in a 96-well XF96 cell culture microplate (Seahorse Biosciences). Prior to the experiments, the culture medium for both cell types was replaced, and the cells were incubated in assay medium for 1 hour at 37°C in a CO2-free incubator. OCR was measured by sequentially injecting 0.5 μM oligomycin (Sigma-Aldrich), 0.5 μM FCCP (carbonyl cyanide-4-(trifluoromethoxy) phenylhydrazone, Sigma-Aldrich), and a mixture of 0.5 μM rotenone/antimycin A (Sigma-Aldrich), with readings taken every 7 minutes. The OCR after FCCP treatment indicates the maximal respiratory capacity of the cells.

### Mitochondrial superoxide detection

Mitochondrial superoxide production was assessed using fluorogenic dye that selectively targets mitochondria and is oxidized by superoxide to emit red fluorescence (ab219943, abcam). TNBC cells (MDA-MB-231) were seeded in 6-well plates or on glass coverslips in 24-well plates and treated as indicated. Following treatment, cells were washed twice with PBS and incubated with 5 µM staining reagent diluted in PBS at 37°C for 10 minutes in the dark. After staining, cells were washed three times with PBS to remove excess dye. For confocal microscopy, stained cells on coverslips were fixed with 4% paraformaldehyde for 15 minutes.

### Tumorigenesis and metastasis assay *in vivo*

For the orthotopic breast cancer model, female NOD/SCID gamma (NSG) mice (4-6 weeks old) were housed at a constant temperature of 22°C on a 12-hour light-dark cycle with access to food and tap water. The NSG mice were randomly divided into four groups: the control group (negative micro-RNA mimics), the miR-6126 mimics group, the miR-6126 + GRP78 cDNA group, and the GRP78 cDNA group. MDA-MB-231 cells (1 x 10^6^) suspended in 100 μl of DMEM-matrigel were injected into the mammary pad of the mice. One week after injection, the mice were injected with either miRNA negative control, miR-6126 mimics, miR-6126 mimics + GRP78 cDNA, or GRP78 cDNA at the tumor cell inoculation sites twice a week. Tumor volume was measured and recorded every third day, and growth curves were plotted based on the tumor volume of each group. Mastectomy was performed after 14 days of treatment, and two weeks after resection, the animals were sacrificed, and the lungs were collected for macro and micrometastasis analysis.

### Statistical analysis

All results will be analyzed using standard statistical techniques. Prism 7.0 (GraphPad) will be utilized for nonlinear regression analysis. The overall significance of means across multiple groups will be assessed by analysis of variance (ANOVA). Data will be analyzed using separate one-way ANOVA tests. Quantitative data will be presented as the mean ± SD based on three or more replicates. A p-value < 0.05 will be considered statistically significant (*), P-value < 0.01 highly significant (**), P-value < 0.001 extremely significant (***), and "ns" will indicate not significant.

## Results

### Expression of miR-6126 is decreased and contribute to cell apoptosis in TNBC cells

**Figure 1:**
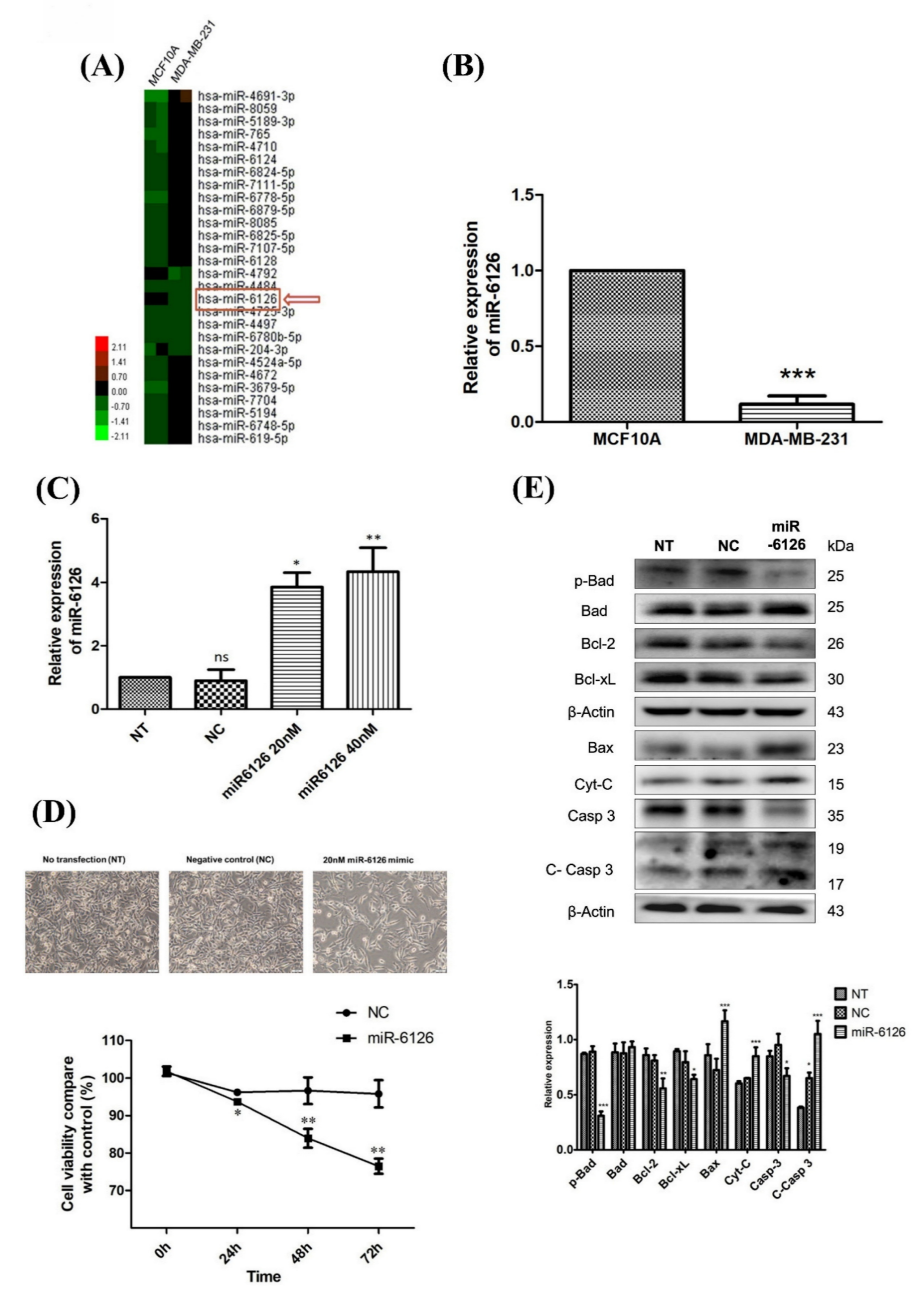
** miR-6126 inhibits cell proliferation and induces apoptosis in triple-negative breast cancer (TNBC) cell lines.** (A) MicroRNA expression in normal breast epithelial cells (MCF10A) and TNBC cell line (MDA-MB-231) was assessed by miRNA microarrays to discover that miR-6126 was downregulated. (B) Analysis results of miR-6126 by qRT-PCR. (C) MDA-MB-231 cells were not transfected (NT), transfected with miRNA mimics negative control (NC) and 20 nM or 40 nM miR-6126 mimics. (D) Cell number was detected after transfection with microscope 100X (Scale bars: 100 μm). Cell viability was determined using the MTT assay. (E) Protein levels determined by Western blotting in MDA-MB-231 cell lines were transfected with miR-6126 mimics. Values shown are means ± SD. Quantification of the result is shown (n=3) ns: no significant, *p < 0.05, **p < 0.01, ***p < 0.001 versus untreated control cells.

To elucidate the role of miRNA in TNBC, we employed miRNA microarray analysis to compare the differential miRNA expression levels between normal breast epithelial cells (MCF10A) and TNBC cells (MDA-MB-231). Our findings revealed that miR-6126 expression was notably reduced in TNBC **(Fig. 1A)**. Quantitative real-time PCR was conducted to assess miR-6126 expression levels in MCF10A and breast cancer cell lines. The results revealed a significant downregulation of miR-6126 in breast cancer cell line MDA-MB-231 compared to MCF10A **(Fig. 1B)**. Furthermore, to elucidate the function of miR-6126 in TNBC, MDA-MB-231 cells were transfected with miR-6126 mimics or a negative control for miRNA mimics. Real-time PCR analysis revealed that miR-6126 expression was significantly elevated in the miR-6126-transfected MDA-MB-231 cells compared to cells with no transfection or those transfected with the negative control **(Fig. 1C)**. Following the overexpression of miR-6126, MDA-MB-231 cells were assessed for viability by using the MTT assay. The results demonstrated that overexpression of miR-6126 decrease the cell viability in TNBC cells **(Fig. 1D)**. The process of programmed cell death, or apoptosis, is typically characterized by alterations in the levels of apoptosis-related proteins 15, 16. And we found that miR-6126 induces apoptosis-relative markers, such as Bax, Cytochrome-c, and cleaved caspase 3 protein expression. Additionally, anti-apoptotic proteins, such as p-Bad, Bcl-2, and Bcl-xL were decrease **(Fig. 1E)**. Collectively, these results suggest that miR-6126 act as a tumor suppressor in TNBC.

### miR-6126 inhibits the proliferation of TNBC cells by directly targeting GRP78

**Figure 2:**
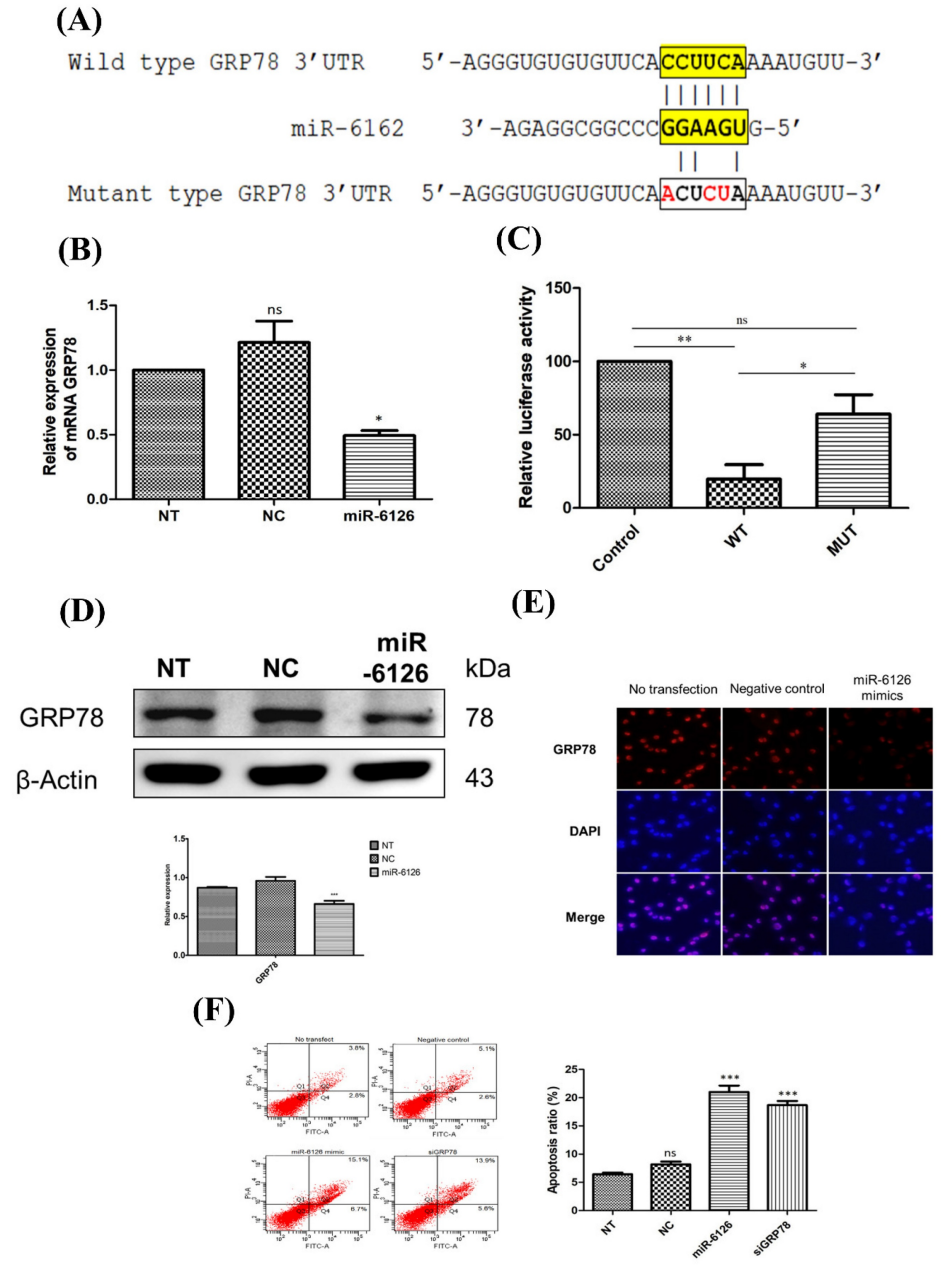
** miR-6126 induces cell apoptosis by inhibiting GRP78 expression.** (A) Schematic showed reporter constructs of wild-type GRP78 3'UTR (upper panel) and GRP78 3'UTR with mutated miR-6126-binding site (lower panel). (B) Analysis of mRNA GRP78 was detected by qRT-PCR. (C) The activity of miR-6126 promotor was analysis by luciferase gene reporter assay. (D) Protein levels of GRP78 was determined by Western blotting. (E) Immunofluorescent staining of GRP78 in MDA-MB-231 cells by fluorescence microscope 100X (Scale bars: 100μm). (F) Apoptosis rate was examined by Annexin-V FITC/PI staining and flow cytometry.

To elucidate the mechanism underlying the abolition of the Warburg effect by miR-6126, we further investigated proteins related to the miR-6126 by using computational prediction algorithms and BLAST alignment on their 3'-untranslated regions (UTR) via Targetscan Human. According to this algorithm, the prediction revealed that the 78 KDa Glucose-Regulated Protein (GRP78), also known as Heat Shock Protein Family A (Hsp70) Member 5 (HSPA5), is a direct target of miR-6126, with a 6-base pairing between complementary sequences on the GRP78 transcript and miR-6126 **(Fig. 2A)**. To verify this targeting, we initially assessed the GRP78 mRNA expression in the MDA-MB-231 cell line following transfection with miR-6126 mimics. Real-time PCR assay revealed a significant reduction in the expression of GRP78 mRNA compared to control mimics and no transfection conditions **(Fig. 2B)**. To further validate that miR-6126 targets GRP78, MDA-MB-231 cells were co-transfected with miR-6126 mimics and constructs containing GRP78, either wild-type or with multiple mutated nucleotides on the miR-6126-binding sites, for luciferase reporter assay. Cells transfected with the wild-type GRP78 construct exhibited an 80% reduction in luciferase activity, whereas no significant reduction was observed with the mutant form **(Fig. 2C)**. Furthermore, the protein level of GRP78 was decreased in MDA-MB-231 cells transfected with miR-6126 mimics, as confirmed by Western blotting and immunofluorescence staining **(Fig. 2D and 2E)**. Furthermore, the data revealed that transfection of miR-6126 and siRNA GRP78 induced apoptosis in TNBC, as examined by flow cytometry **(Fig. 2E)**. Furthermore, flow cytometric analysis of Annexin V and PI stained cells transfected with negative microRNA, miR-6126, or siRNA GRP78 showed that miR-6126 overexpression caused a significant increase in the percentage of apoptosis, from 6.43±0.28 to 21±1.13, and 18.6±0.73 after transfection with siRNA GRP78 **(Fig. 2F)**. Taken together, the results demonstrated that miR-6126 directly binds to the 3'UTR of GRP78, resulting in negative regulation of GRP78 in TNBC cells.

### miR-6126 reduces Warburg effect via GRP78/AKT axis inhibition

**Figure 3:**
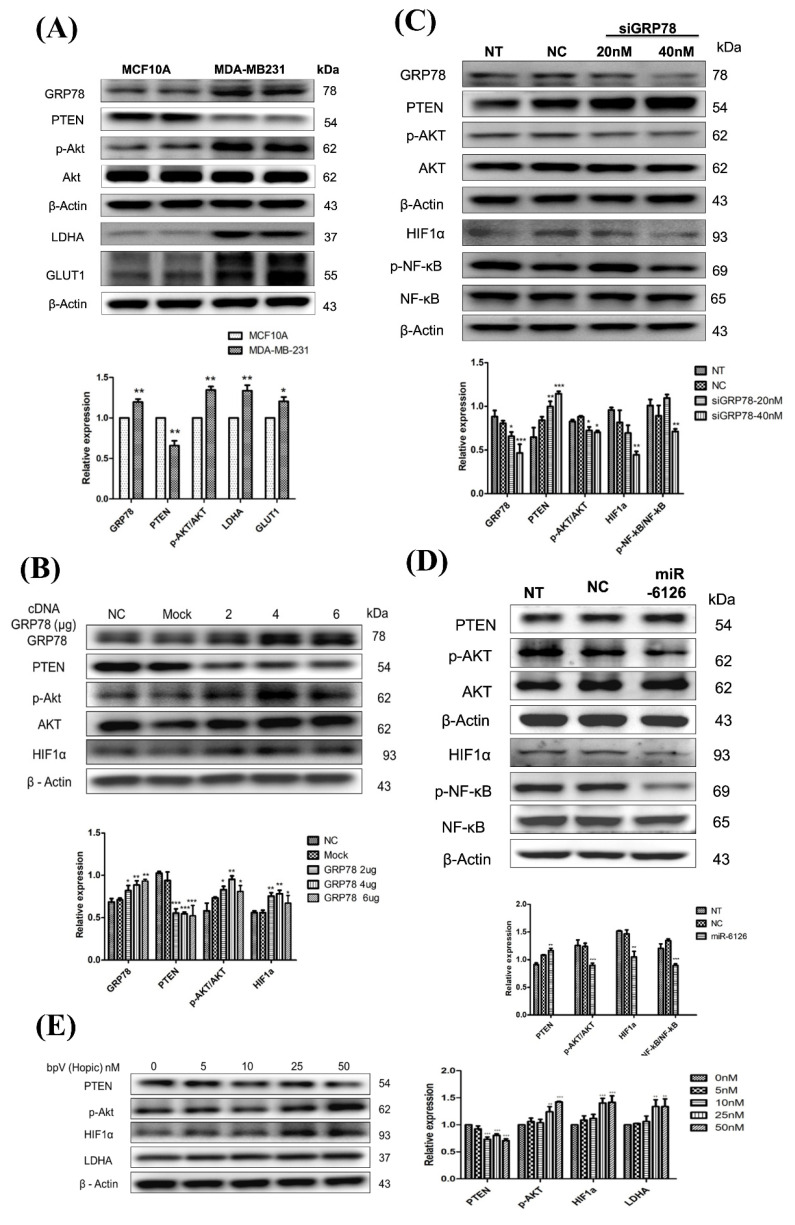
** miR-6126 reduces the PTEN-inhibited Warburg effect by targeting GRP78.** (A) The comparison of protein levels determined by Western blotting in MCF-10A and MDA-MB-231 cell lines. β-actin was served as a loading control. (B) MDA-MB-231 cells were not transfected (NC), transfected with empty plasmid (Mock), 2 μg, 4 μg or 6 μg cDNA GRP78. Protein levels were determined by western blotting. β-actin were served as a loading control. (C) MDA-MB-231 cells were transfected siGRP78 with the indicated concentration. Protein levels were determined by western blotting. (D) MDA-MB-231 cells were not transfected (NT), transfected with miRNA mimics negative control (NC) or 20 nM miR-6126 mimics. Protein levels were determined by Western blotting.

GRP78 has been implicated in promoting glycolysis in cancer cells by stabilizing and activating key glycolytic enzymes and glucose transporters, such as GLUT1 17. Therefore, to assess Warburg effect activity in TNBC, the protein levels of glucose transporter 1 (GLUT1) and lactate dehydrogenase A (LDHA) in MDA-MB-231 were investigated and compared with normal cells MCF10A. The results revealed significantly elevated expression levels of GLUT1 and LDHA proteins in MDA-MB-231 cells compared to MCF10A cells **(Fig. 3A)**. Additionally, it has been reported that the activation of Akt can promote aerobic glycolysis and increase glucose dependence in cancer cells 18. Moreover, PTEN plays a critical role in the aerobic glycolysis of cancer by acting as a negative regulator of Akt phosphorylation. It catalyzes the dephosphorylation of PIP3, thereby inhibiting Akt activation and subsequently suppressing aerobic glycolysis 19. In the MDA-MB-231 cell line, the level of phosphorylated Akt was increased, while PTEN expression was lower than in normal cells. **(Fig. 3A)**. Interestingly, the expression of GRP78 in MDA-MB-231 was also higher than in MCF10A** (Fig. 3A)**, which could be associated with facilitating cell adaptation to stress for survival through the PI3K/Akt pathway 20, 21. Taken together, these data suggest that lower miR-6126 expression may be associated with oncogenic effects on aerobic glycolysis.

Furthermore, to identify the underlying mechanisms of GRP78/AKT axis, we overexpressed GRP78 and showed facilitated p-AKT activation and the downstream HIF1α expression. In contrast, transfection with GRP78 siRNA significantly inhibited PI3K/AKT pathway and the downstream signaling HIF-1α/p-NF-κB expression **(Fig. 3B, 3C)**. To further analyze the activation of downstream signaling pathways mediated by PTEN and induced by miR-6126, the PI3K/AKT signaling pathway was investigated. The results demonstrate that induction of miR-6126 led to downregulation of p-AKT, inhibition of HIF-1α, and inhibition of p-NF-κB **(Fig. 3D)**. To confirm PTEN inhibition in MDA-MB-231 cells and monitor downstream effectors of PTEN-dependent signaling, such as the PI3K/AKT pathway and the Warburg effect, a bisperoxovanadium inhibitor of protein phosphotyrosine phosphatases (bpV(Ohpic)), selective for PTEN, was applied. Elevated activity of phospho-AKT increased HIF-1α and LDHA expression following bpV(Ohpic) treatment in TNBC **(Supplementary Fig. 1)**. These results highlight that miR-6126 inhibits the tumorigenesis of TNBC by enhancing PTEN activation, which negatively regulates the PI3K/AKT pathway.

### miR-6126 inhibit Warburg effect in TNBC cells

**Figure 4:**
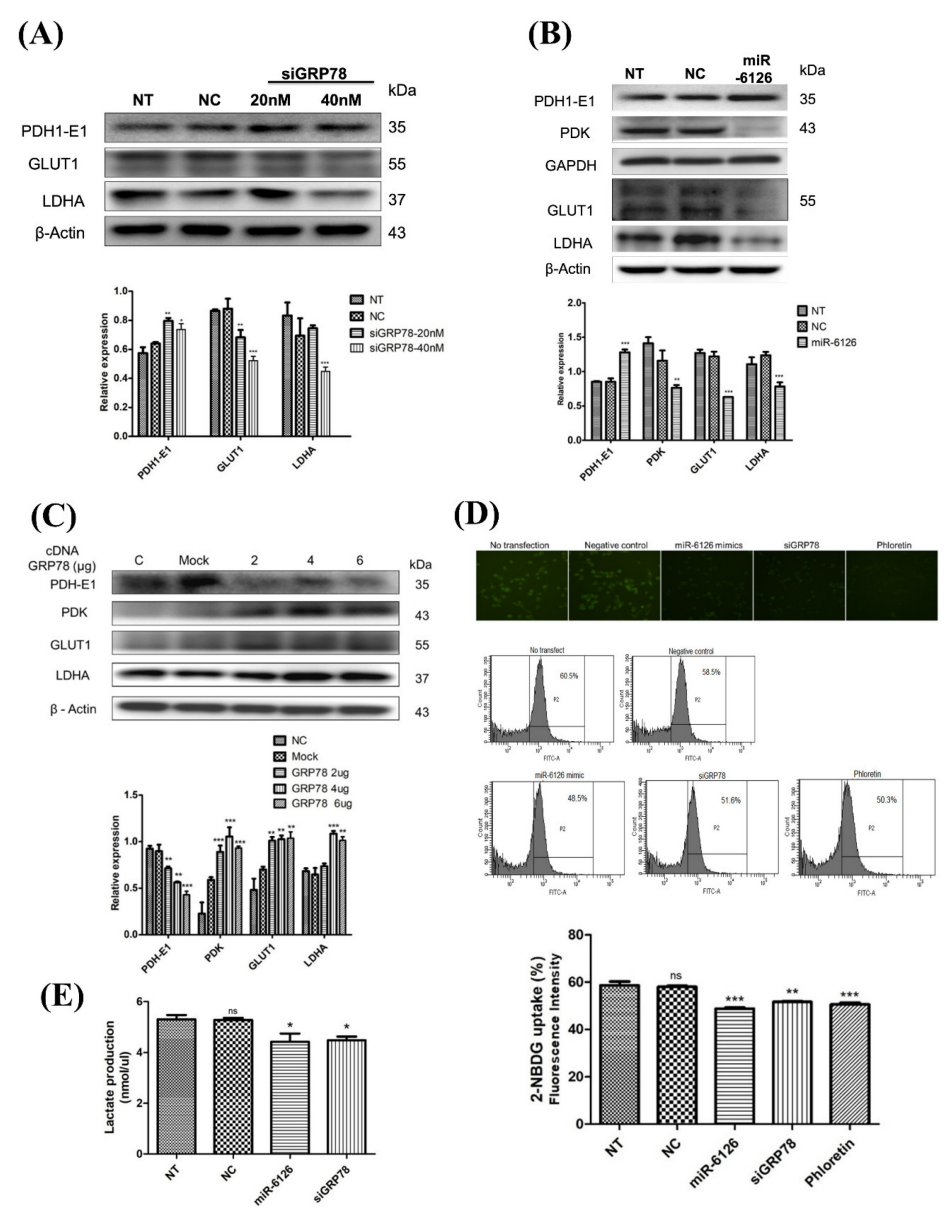
** miR-6126 suppresses the glycolysis pathway in triple-negative breast cancer (TNBC) cell line.** (A) MDA-MB-231 cells were not transfected (NT), transfected with siRNA negative control (NC) or siGRP78. Protein levels were determined by Western blotting. (B) MDA-MB-231 cells were transfected with miRNA mimics negative control (NC) or 20 nM miR-6126 mimics. Protein levels were determined by Western blotting. β-actin and GADPH were served as a loading control. (C) MDA-MB-231 cells were transfected with cDNA negative control (Mock) or 2 μg, 4 μg or 6 μg cDNA of GRP78. Protein levels were determined by Western blotting. (D) MDA-MB-231 cells were transfected with miR-6126 mimics or siGRP78, then incubated with a fluorescent glucose derivative, 2-NBDG, for 30 min before examination by fluorescence microscope 100X (Scale bars: 100μm). Fluorescence intensity of 2-NBDG uptake was analysed by flow cytometry. Phloretin was used as a natural phenol that inhibits glucose uptake. (E) Lactate production was performed by using Lactate Colorimetric/Fluorometric Assay Kit. Values shown are means ± SD. Quantification of the result is shown (n=3) ns: no significant, *p < 0.05, **p < 0.01, ***p < 0.001 versus untreated control cells.

To investigate whether miR-6126 exerts an inhibitory effect on the Warburg effect by targeting GRP78, we suppressed GRP78 or overexpressed miR-6126 expression in MDA-MB-231 cells through transfection with GRP78 siRNA or miR-6126 mimics. This suppression of GRP78 resulted in decreased protein levels of glucose metabolism-related proteins, such as GLUT1 and LDHA, and increased expression of Pyruvate Dehydrogenase E1 (PDH-E1) **(Fig. 4A)** 22. Conversely, overexpression of miR-6126 inhibited the expression of pyruvate dehydrogenase kinase (PDK), GLUT1, and LDHA, while increasing the expression of PDH-E1 in TNBC cells **(Fig. 4B)**. Furthermore, the overexpression of GRP78 significantly increased the expression of Warburg effect-related signaling proteins, including GLUT1, LDHA, and PDK, while reducing the expression of PDH-E1 **(Fig. 4C)**. In addition, following transfection with miR-6126 or GRP78 siRNA, glucose uptake and lactate production were decreased, as detected by fluorescence microscopy and flow cytometry **(Fig. 4D, 4E)**. After incubation with a fluorescent glucose derivative, 2-NBDG, and using Phloretin as an inhibitor of glucose uptake, the data indicate that miR-6126 inhibits the Warburg effect by targeting GRP78.

### Restoration of mitochondrial function as a target for miR-6126

**Figure 5:**
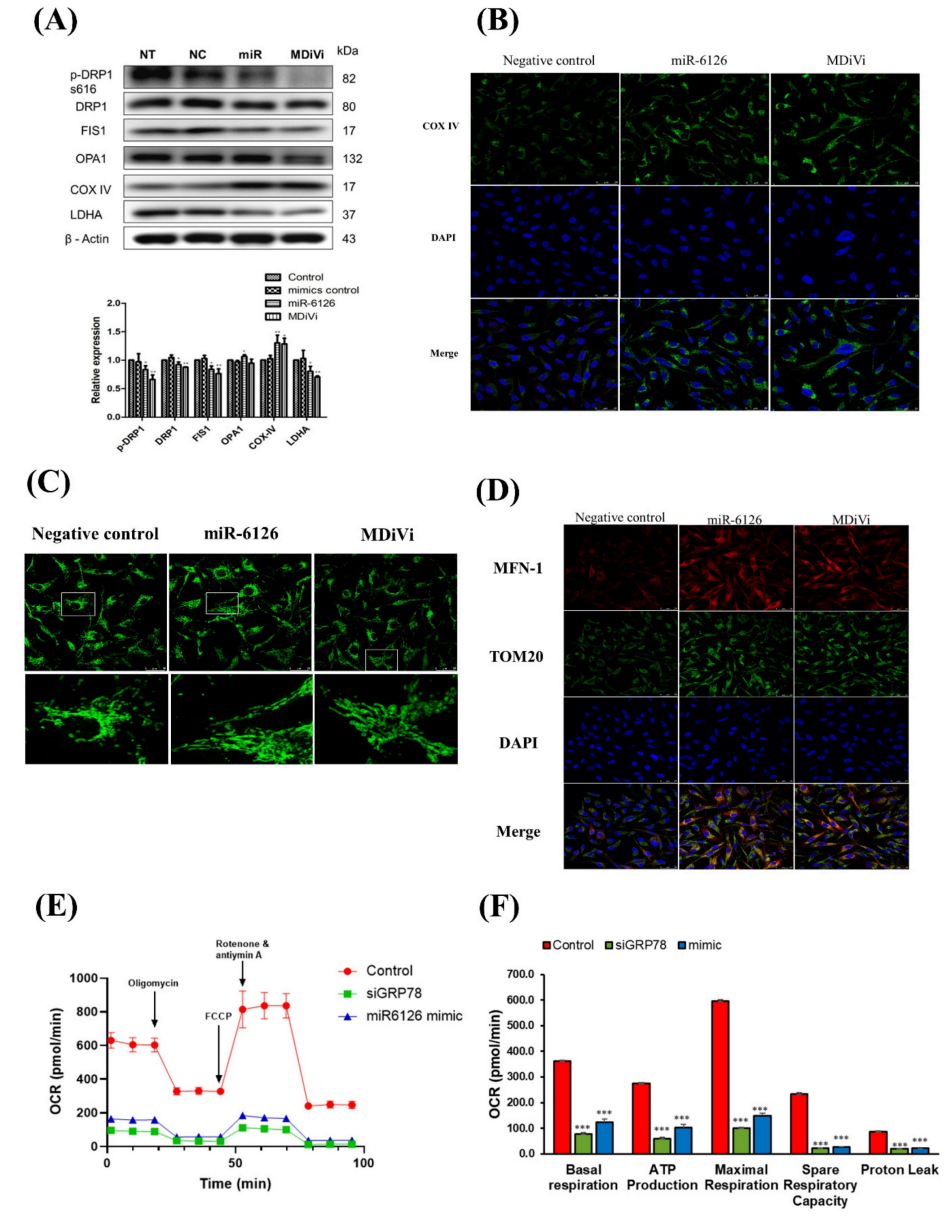
** miR-6126 and knockdown of GRP78 suppress mitochondrial function by inhibiting mitochondrial fission in triple-negative breast cancer (TNBC) cell line.** (A) MDA-MB-231 cells were not transfected (NT), transfected with miRNA mimics negative control (NC), 20 nM miR-6126 mimics, or 100 μM MdiVi-1 for 24 h. Protein levels were determined by Western blotting. (B) Immunofluorescent staining of COX IV in MDA-MB-231 cells with miR-6126 mimics transfection or 100 μM MdiVi-1 treatment (Scale bars: 25 μm). (C) Mitochondria were labelled by using an anti-TOM20 antibody (Scale bars: 25 μm). (D) Immunofluorescent staining of MFN-1 and TOM20 in MDA-MB-231 cells with miR-6126 mimics transfection or 100 μM MdiVi-1 treatment (Scale bars: 25 μm). (E) The mitochondrial respiration profile was analyzed by measuring OCR in MB231 cells transfected with GRP78 siRNA (siG) and the miR-6126 mimic. (F) Quantification of basal respiration, ATP production, maximal respiration, spare respiratory capacity, and proton leak in MB231 cells transfected with GRP78 siRNA and the miR-6126 mimic. Values shown are means ± SD. Quantification of the result is shown (n=3) *p < 0.05, **p < 0.01 and ***p < 0.001versus untreated control cells.

Recent studies have increasingly demonstrated that mitochondrial dynamics significantly influence metabolic phenotypes, including the Warburg effect, in cancer cells 23, 24. However, it is still unknown whether miR-6126 inhibits the Warburg effect by regulating mitochondrial dynamics. Therefore, to investigate the role of miR-6126 in mitochondrial function, we treated MDA-MB-231 cells with mitochondrial division inhibitor 1 (Mdivi-1), a specific inhibitor of dynamin-related protein 1 (Drp1), and compared this with the effects of miR-6126 overexpression. As shown in **Figure 5A**, increasing the concentration of Mdivi-1 significantly reduced levels of LDHA, suggesting that inhibiting mitochondrial fission can also suppress the Warburg effect in TNBC. Furthermore, overexpression of miR-6126 resulted in the downregulation of LDHA, phospho-Drp1 (Ser616) and FIS1, while OPA1 and COX IV was upregulated **(Fig. 5A and 5B)**, which were already known to play a critical role in mitochondria dynamics regulation 25. In addition, confocal microscopy was used to further investigate the effect of miR-6126 on mitochondrial dynamics, with mitochondrial morphology labeled using an anti-TOM20 antibody (Green). After transfection with miR-6126 mimics, MDA-MB-231 cells exhibited elongated mitochondria, while cells treated with the miRNA negative control showed fragmented mitochondria morphology **(Fig. 5C)**. Furthermore, Mitofusin-1 (MFN-1) has been demonstrated to play a crucial role in mitochondrial dynamics by promoting fusion 26. To confirm the expression levels of MFN-1 in TNBC cells following miR-6126 overexpression, immunofluorescence staining was performed. Representative images from miR-6126 mimic transfection showed a similar increase in MFN-1 levels as observed with Mdivi-1 treatment **(Fig. 5D)**. These results support that miR-6126 impair mitochondria function by inhibiting fission and promoting fusion in TNBC cells. To further investigate whether GRP78 and miR-6126 affect the mitochondria-dependent metabolic activity of cells, we assessed mitochondrial respiration using the Seahorse assay in MDA-MB-231 cells. We found that siGRP78 and the miR-6126 mimic decreased basal respiration, ATP production, maximal respiration, spare respiratory capacity, and proton leak in the cells **(Fig. 5E and 5F)**. These results suggest that knocking down GRP78 or overexpressing miR-6126 can reduce the energy metabolism requirements of TNBC cells.

### Inhibition of GRP78 sensitizes TNBC Cells to doxorubicin by inducing mitochondrial ROS and suppressing AMPK activity

**Figure 6:**
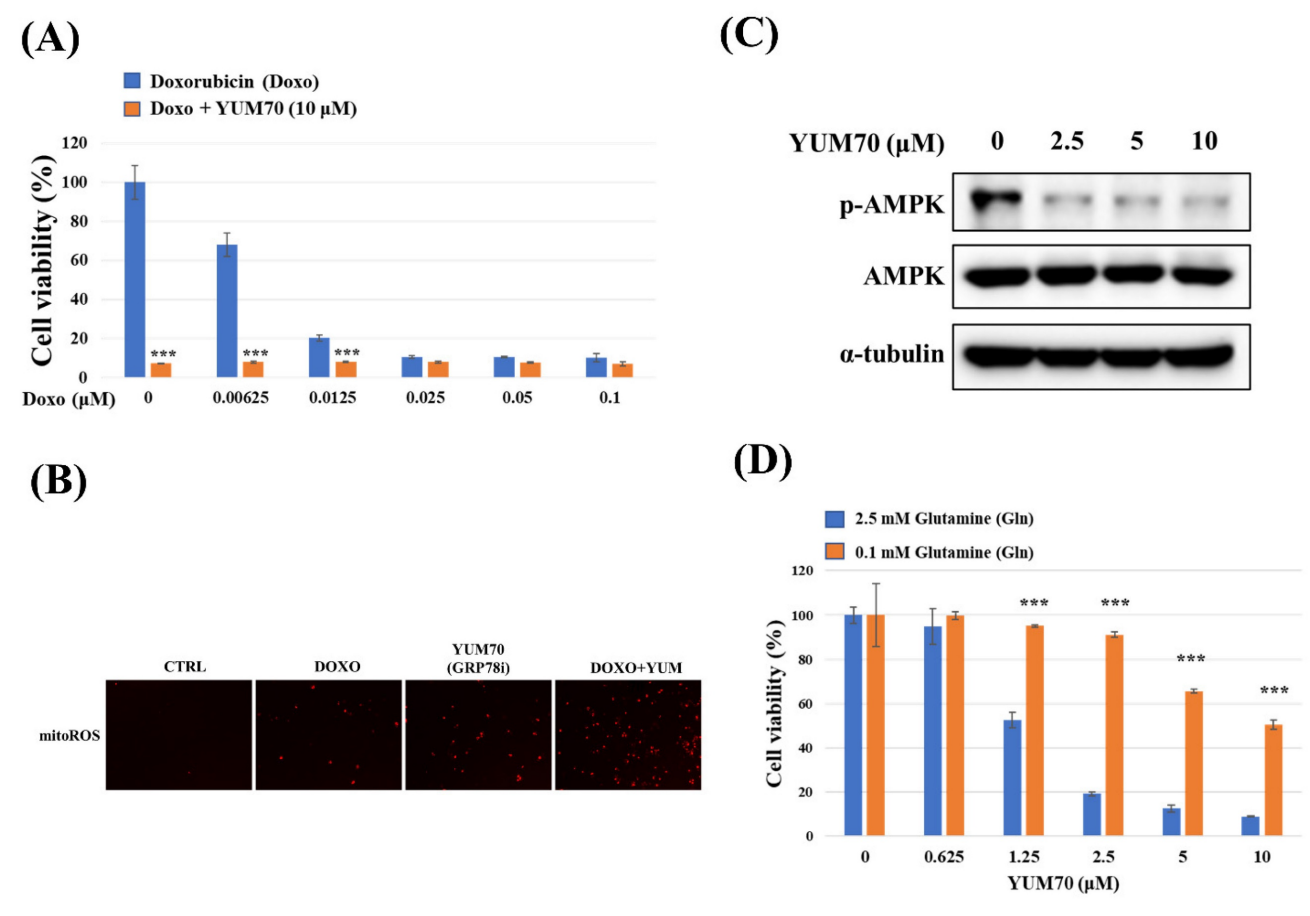
** GRP78 inhibition enhances doxorubicin (Doxo) cytotoxicity and modulates glutamine-dependent cell viability in triple-negative breast cancer (TNBC) cells.** (A) MDA-MB-231 cells were treated with increasing concentrations of Doxo alone or in combination with the GRP78 inhibitor YUM70 (10 µM) for 24 hours. Cell viability was assessed using the MTT assay. (B) Representative images of mitochondrial ROS (mitoROS) detected by mitochondria superoxide detection staining after treatment with Doxo (0.005 µM), YUM70 (10 µM), or the combination for 24 hours. Increased mitoROS was observed in the combination group, indicating enhanced oxidative stress. (C) Western blot analysis showing the effect of YUM70 on AMPK activation. MDA-MB-231 cells were treated with increasing concentrations of YUM70 (0-10 µM) for 24 hours. Phosphorylation of AMPK (p-AMPK) and total AMPK were assessed; α-tubulin served as a loading control. (D) Cell viability of MDA-MB-231 cells treated with YUM70 under high (2.5 mM) or low (0.1 mM) glutamine conditions for 24 hours. YUM70 treatment significantly reduced cell viability in glutamine-replete conditions, suggesting that GRP78 inhibition affects glutamine-dependent metabolic survival. Data are presented as mean ± SD; ***p < 0.001 vs. untreated control.

To investigate the therapeutic potential of targeting GRP78 in combination with standard chemotherapy, we first assessed the effect of the GRP78 inhibitor YUM70 on doxorubicin-induced cytotoxicity in MDA-MB-231 cells. As shown in **Figure 6A**, co-treatment with YUM70 significantly enhanced the anti-proliferative effect of doxorubicin at all tested concentrations compared to doxorubicin alone, indicating a synergistic inhibitory effect on cell viability. We next examined whether the combination treatment induced oxidative stress in mitochondria. Mitochondria superoxide detection staining revealed that mitochondrial ROS (mitoROS) levels were markedly increased upon combined treatment with doxorubicin and YUM70, compared to either agent alone **(Fig. 6B)**. This suggests that enhanced mitochondrial oxidative stress contributes to the observed cytotoxic synergy. Given the critical role of AMPK in metabolic stress responses 27, we examined whether GRP78 inhibition modulates AMPK signaling. Western blot analysis demonstrated that YUM70 treatment led to a dose-dependent decrease in AMPK phosphorylation **(Fig.6C)**, indicating suppression of AMPK activity upon GRP78 inhibition. Finally, we explored whether GRP78 regulates cell viability through glutamine metabolism. MDA-MB-231 cells were treated with increasing doses of YUM70 under high (2.5 mM) or low (0.1 mM) glutamine conditions. As shown in **Figure 6D**, GRP78 inhibition significantly reduced cell viability in glutamine-rich medium, but had minimal impact under glutamine-depleted conditions. These findings suggest that GRP78 supports TNBC cell survival, in part, through the regulation of glutamine-dependent metabolic pathways.

### miR-6126 inhibits tumorigenesis and metastasis of TNBC *in vivo*

**Figure 7:**
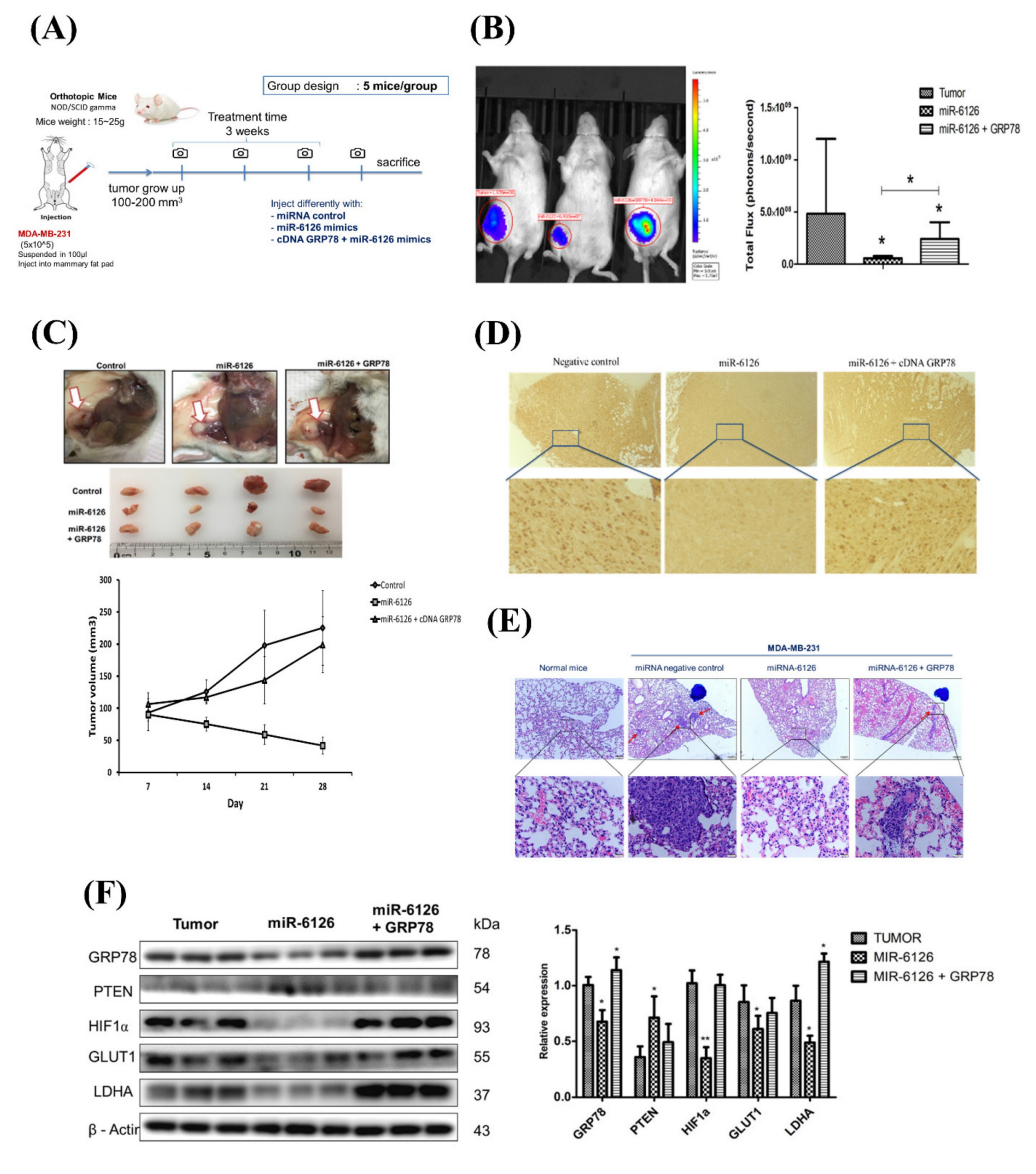
** miR-6126 inhibits tumor growth and metastasis in an orthotopic breast cancer mouse model.** (A) The experiment design for evaluating the inhibitory effects of miR-6126 on tumorigenesis and metastasis of TNBC *in vivo* (B) To monitor primary tumor growth after one-week injection, luciferase substrate luciferin was injected, and bioluminescence emission was measured using *in vivo* imaging. Mice with luciferase-expressing MDA-MB-231-Luc breast cancer cells implanted in their mammary fat pat. Bioluminescent signals photons/second were quantified using the IVIS imaging system. (C) Tumor volume was recorded weekly after reaching a size of approximately 100 mm^3^ (D) Representative images of IHC staining of ki-67 in tumor sample using microscope 100X (Scale bars: 100μm). (E) Effect of miR-6126 on inhibition of breast cancer metastasis in vivo. Histological (H&E) staining performed in lung tissue following the different treatments compare with normal mice (Scale bars: 50 μm). (F) Protein level analysis from tumor samples by Western blotting. Values shown are means ± SD. Quantification of the result is shown (n=3) *p < 0.05, **p < 0.01 and ***p < 0.001 versus untreated control cells.

**Figure 8:**
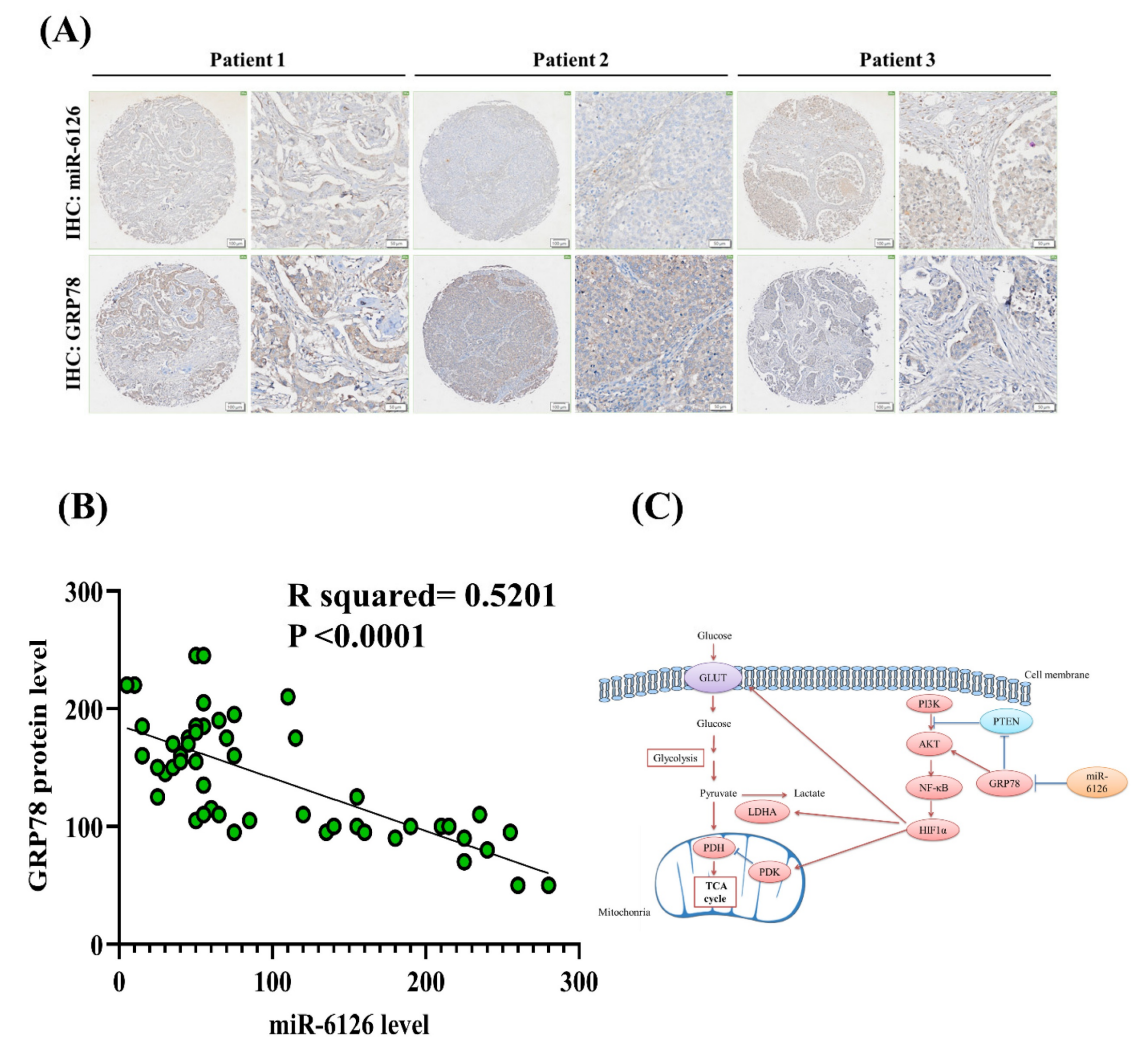
** MiR-6126 expression is correlated with GRP78 in human triple-negative breast cancer (TNBC) tumor tissue.** (A) Representative images showing immunohistochemical staining of miR-6126 and GRP78 in human TNBC tumor tissues. Scale bars range from 50 to 100 μm. (B) Correlation analysis between miR-6126 and GRP78. Sample size (n) = 50. Statistical calculations were performed on biological replicates using simple linear regression. Expression levels were normalized to the H-score. (C) Proposed mechanism by which miR-6126 enhances PTEN activation through GRP78 inhibition, leading to the reduction of the Warburg effect and mitochondria fission in TNBC.

Based on the inhibitory role of miR-6126 in the Warburg effect in vitro. Next, we explored its suppressive effects on tumorigenesis and metastasis *in vivo*, as well as its impact on GRP78 expression, using an orthotopic breast cancer model. MDA-MB-231 cells were injected into the mammary fat pad of female NOD/SCID gamma (NSG) mice to initiate tumorigenesis **(Fig. 7A)**. Primary tumor growth was monitored one week after injection by administering the luciferase substrate luciferin and detecting bioluminescence emission using *in vivo* imaging. The results showed that overexpression of miR-6126 decreased tumor growth and the proliferation marker Ki-67 expression, while GRP78 reversed the inhibitory effect of miR-6126 in the mouse model **(Fig. 7B-D)**. Moreover, we observed that miR-6126 also reduced the lung metastasis of MDA-MB-231 cells originating from the breast, as confirmed by histological (H&E) staining **(Fig. 7E and Supplementary Table 1)**. Furthermore, protein expression levels in the tumor tissue revealed that miR-6126 inhibits the expression of Warburg effect-related signaling proteins such as GRP78, HIF1α, GLUT1, and LDHA but induces PTEN expression. In contrast, overexpression of GRP78 impairs the effect of miR-6126 on these protein expression levels **(Fig. 7F)**. In addition, to identify the correlation between miR-6126 and GRP78 in human tumor tissue, we performed in situ hybridization (ISH) and immunohistochemistry (IHC) to detect the expression levels of miR-6126 and GRP78 in the tumor tissue microarray. In clinical TNBC samples, a significant inverse correlation was observed between miR-6126 and its target GRP78 **(Fig. 8A-B and Table 1)**. These results demonstrate that miR-6126 suppresses tumor growth and metastasis in TNBC and indicate that miR-6126 can attenuate the Warburg effect and mitochondrial function by targeting GRP78 **(Fig. 8C)**.

## Discussion

Recently, there has been renewed interest in targeting and restoring mitochondrial homeostasis and reducing the Warburg effect as promising strategies for cancer control and management in TNBC. In this study, we demonstrated that inhibiting the aerobic glycolysis pathway through the downregulation of GLUT1 and LDHA expression via miR-6126/GRP78 axis, and restoring mitochondrial function by reducing Drp1 expression to promote mitochondrial fusion, represents a viable therapeutic approach for treating TNBC.

Half of the human miRNAs are implicated in cancer, with their expression levels often altered in cancer patients, influencing disease progression and severity 28, 29. In breast cancer, miRNAs play crucial roles in tumorigenesis regulation, making them promising therapeutic targets 30, 31. For instance, Nassirpour et al. found high levels of miR-221 in TNBC, and its inhibition led to cell cycle arrest, apoptosis, and suppressed tumor growth 32. Similarly, miR-205 acts as a tumor suppressor, downregulated in TNBC, and is regulated by the PTEN gene 33. Another tumor suppressor miRNA, miR-206, inhibits cell growth, migration, and invasiveness, and its upregulation impedes metastasis. Moreover, miR-206 is involved in apoptosis, and its downregulation in breast cancer correlates with advanced disease stages and shorter overall survival 34. Here, we identify miR-6126 as a novel tumor-suppressive miRNA scarcely reported in TNBC. In this study, we observed downregulation of miR-6126 in TNBC using the MDA-MB-231 cell line. Furthermore, our findings showed that overexpression of miR-6126 promoted apoptosis in TNBC. Additionally, another study suggested that ovarian cells release miR-6126 through exosomes, contributing to tumorigenesis, metastasis, and multidrug resistance 7.

Moreover, miR-6126 has demonstrated tumor suppressive activity in other cancer types. In ovarian cancer, miR-6126 was identified as a component of tumor-derived exosomes that suppress angiogenesis and metastasis 7. Its reported targets include genes involved in cytoskeletal remodeling and chemoresistance, implicating it in modulating tumor-stroma interactions and drug response. Given that GRP78 is a shared oncogenic hub in multiple cancers, and miR-6126 appears to regulate distinct oncogenic networks. Based on bioinformatics analyses, we further predicted GRP78 as a target of miR-6126. While our study primarily focuses on GRP78 as a direct functional target of miR-6126 in TNBC, emerging evidence suggests that miR-6126 may have a broader impact across multiple cancer subtypes via other molecular targets. For instance, previous study identified integrin-β1 (ITGB1) as a direct target of miR-6126 in ovarian cancer 7. The suppression of ITGB1 by miR-6126 led to downstream inhibition of paxillin phosphorylation and impairment of metastatic properties such as cell migration, invasion, and angiogenesis. Their reverse-phase protein array (RPPA) and Western blot analysis also revealed that miR-6126 overexpression reduced PI3K/AKT pathway signaling in multiple ovarian cancer cell lines. Furthermore, miR-6126 was implicated in modulating DNA damage repair pathways and tumor suppressor mechanisms in colorectal cancer, particularly through regulation of mismatch repair gene expression 35. These findings underscore the broader tumor-suppressive potential of miR-6126 across various malignancies beyond TNBC.

GRP78 plays crucial roles in enhancing cell proliferation and protecting against microenvironmental stress associated with tumorigenesis. These functions of GRP78 contribute significantly to the activation of AKT signaling 36. Additionally, as an ER Ca^2+^ binding protein, GRP78 maintains protein quality control and serves to uphold homeostasis 37. GRP78, with its anti-apoptotic features, is known to inhibit the proapoptotic BH3 protein BIK and is implicated in the progression of human breast and colorectal cancers 38, 39. Moreover, it has been identified that GRP78 may be associated with the repression of caspase-7 40, contributing to the elevation of cancer cells escaping the immune system 41. Interestingly, the upregulation of GRP78 was reported as a strong initiating force for tumor development in prostate epithelium with PTEN deficiency 21. This suggests that the inhibition of GRP78 may potently suppress tumorigenesis by inactivating the PI3K/AKT survival pathway. In addition, GRP78 has been shown to be negatively regulated by the tumor suppressor BRCA1 in ovarian and breast cancer cells, while mutant BRCA1 enhances GRP78-mediated cell survival and resistance to apoptosis 42. Unfortunately, it has been histologically and transcriptionally shown that dysfunction of BRCA1 is common in TNBC 43, and more than 80% of breast cancers in women who carry germ-line BRCA1 mutations are triple-negative 44. In addition, as a central regulator of the unfolded protein response (UPR), GRP78 is upregulated in various malignancies, including lung adenocarcinoma 45, prostate 46 and ovarian cancers 47, where it promotes tumor growth, stress tolerance, and resistance to apoptosis. In hepatocellular carcinoma, GRP78 expression is tightly linked to metastatic progression and resistance to conventional therapies 48. Therefore, targeting GRP78 could be a promising therapeutic strategy for BRCA1-mutant TNBC. Furthermore, our findings reveal that the protein expression level and mRNA of GRP78 were downregulated by miR-6126 overexpression. In addition, luciferase reporter assays demonstrated that the 3'UTR region of GRP78 can be targeted by miR-6126. Surprisingly, miR-6126 mimics treatment led to a decrease in the metabolic pathway of the Warburg effect and negatively regulated LDHA, GLUT1, PDK, as well as activated PDH-E1.

In addition to inhibiting the Warburg effect, restoring mitochondrial steady state is another function of miR-6126 for cancer control and management. It has been reported that mitochondria play a critical role in response to physiological or stress stimuli by controlling their structure and function 49. Mitochondrial dynamics involve in the processes of fission (fragmentation) and fusion (elongation), which could be regulated by the level of Drp-1 phosphorylation and MFN-1 protein 50. Mitochondrial fission or fragmentation often exhibits in cancer cells. Furthermore, low expression or inhibition of MFN1, as well as upregulation of Drp1, enhance this phenotype in metastatic breast cancer 51, colorectal cancer cells 40, lung cancer 52, melanoma 53, glioblastoma 54, pancreatic cancers 55, and neuroblastoma 56. Our studies provide evidence that miR-6126 upregulates mitochondrial fusion while downregulating fission, leading to a decrease in phospho-Drp-1 and FIS1 expression, as well as an enhancement of OPA1 expression. Accordingly, the results of immunofluorescence analysis showed that MNF-1 was increased following treatments with miR-6126 overexpression or mitochondrial division inhibitor 1 (MdiVi-1), an inhibitor of dynamin-related protein 1 (Drp1). Surprisingly, mitochondrial fission inhibition has been shown to enhance respiratory complex IV in TNBC following miR-6126 mimics treatments. Loss of complex IV in cancer cells has been reported to be associated with downregulation of electron transport, OXPHOS attenuation, decreased reactive oxygen species (ROS) production, ultimately resulting in loss of mitochondrial function and enhanced cancer cell survival 57-59. These observations provide strong evidence that downregulation of miR-6126 can enhance mitochondrial fission and dysfunction, leading to increased cancer growth in TNBC.

## Conclusion

In summary, the current study demonstrates that miR-6126 can suppress the Warburg effect, leading to cell apoptosis by decreasing cellular GRP78 levels and enhancing PTEN activation in TNBC. Conversely, overexpression of GRP78 significantly increased levels of LDHA, GLUT1, and p-AKT. Additionally, miR-6126 can restore mitochondrial function through elevated mitochondrial fusion. Therefore, miR-6126, as a tumor suppressor, represents a potential therapeutic target in TNBC treatment.

## Supplementary Material

Supplementary figures and tables.

## Figures and Tables

**Table 1:** The correlation of miR-6126 and GRP78 in human Triple-negative breast cancer (TNBC)

Parameter	Expression status	Case no.	GRP78 expression		*P* value	miR-6126 expression		*P* value
			Low	High		Low	High	
GRP78	Low expression	2				0	2	
	High expression	48				26	22	< 0.01
miR-6126	Low expression	26	0	26				
	High expression	24	2	22	< 0.01			
