# Supplementary material for: miR-6126 modulates GRP78 to suppress the Warburg effect and mitochondrial dynamics in triple-negative breast cancer
Source: Int J Med Sci. 2025 Jul 28;22(14):3598–616. doi: 10.7150/ijms.107240 (PMC12434812; doi:10.7150/ijms.107240)
Supplement: Supplementary file 1 — Supplementary figures and tables. [file ijmsv22p3598s1.pdf]

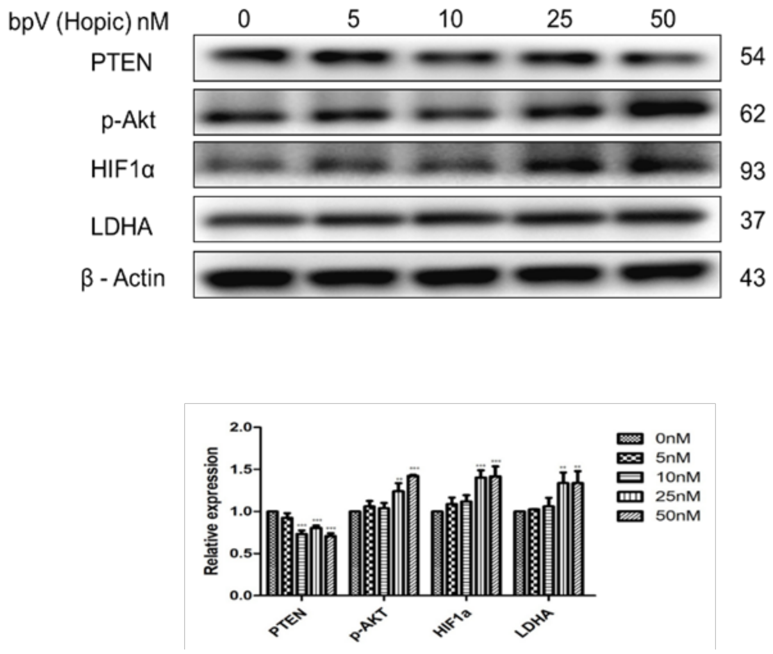

Supplementary Fig 1. MDA-MB-231 cells were treated phospho-tyrosine phosphatase inhibitor Bpv (Hopic) 5 nM, 10 nM, 25 nM or 50 nM for 24 h. Protein levels were determined by Western blotting. β-actin were served as a loading control.Values shown are means ± SD. Quantification of the result is shown (n=3) *p < 0.05, **p < 0.01, ***p < 0.001 versus untreated control cells.

Supplementary Table 1. Approximate lung, liver and brain metastasis MDA-MB-231 cells were quantified by qPCR determination of human ALU loci in mouse lung, liver and brain tissues.

Approximate lung, liver and brain metatasis MDA-MB-231 cells were quantified by **qPCR** determination of **human ALU loci** in mouse lung, liver and brain tissues.

**Ct Mean**	**LUNG**	**LIVER**	**BRAIN**
Tumor	13.224	16.434	20.158
Tumor	12.874	15.168	20.418
Tumor	10.791	15.311	19.407
miR-6126	Undetermined	Undetermined	Undetermined
miR-6126	Undetermined	Undetermined	Undetermined
miR-6126	Undetermined	Undetermined	Undetermined
miR-6126 + GRP78	14.988	16.689	Undetermined
miR-6126 + GRP78	17.806	17.227	Undetermined
miR-6126 + GRP78	15.277	16.399	Undetermined
